# Prevalence and correlates of ADHD among adolescents in a Beirut community sample: results from the BEI-PSY Study

**DOI:** 10.1186/s13034-017-0156-5

**Published:** 2017-04-17

**Authors:** Elias Ghossoub, Lilian A. Ghandour, Fadi Halabi, Pia Zeinoun, Al Amira Safa Shehab, Fadi T. Maalouf

**Affiliations:** 1 Department of Psychiatry, American University of Beirut, P.O. Box 11-0236, Riad El-Solh/Beirut, 1107 2020 Lebanon; 2 Department of Epidemiology and Population Health, Faculty of Health Sciences, American University of Beirut, Beirut, Lebanon; 3 Department of Psychiatry, Washington University in St. Louis, St. Louis, USA; 4 Department of Psychology, Faculty of Arts and Sciences, American University of Beirut, Beirut, Lebanon; 5 Department of Psychology, Queens College, City University of New York, New York, USA

**Keywords:** Attention deficit disorder with hyperactivity, Epidemiology, Lebanon, Patient acceptance of health care

## Abstract

**Background:**

This study aims to investigate the prevalence, correlates and treatment seeking behavior related to ADHD among adolescents from Lebanon.

**Methods:**

Five hundred and ten adolescents were recruited through multistage stratified cluster sampling of households in Beirut, and separately interviewed along with one parent/legal guardian, using the DAWBA. All adolescents completed the PRQ and the SDQ; the parent/legal guardian also completed the SDQ and provided basic demographic information, including attitudes towards seeking mental health services.

**Results:**

10.20% of the adolescents were diagnosed with ADHD. Having ADHD was associated with having academic difficulties and being involved in bullying. Adolescents with ADHD also had higher odds of drinking alcohol, smoking cigarettes, and having comorbid emotional and conduct disorders (compared to those without ADHD). Adolescents with ADHD and their parents reported a higher burden of illness and were more likely to consider seeing a mental health professional than healthy adolescents and their parents.

**Conclusion:**

ADHD among adolescents in Lebanon warrants closer attention, mainly increased awareness in the larger public, and stronger commitment to increase treatment resources to the community.

## Background

Attention-deficit/hyperactivity disorder (ADHD) is a neurodevelopmental disorder with a triad of symptom clusters: inattention, hyperactivity and impulsivity [1]. Symptoms need to be present in two settings at least and have to cause significant distress and functional impairment [2]. The *Diagnostic and Statistical Manual*—*5th edition* (DSM-V) recently updated the cut-off age of onset to be below 12 years of age [1], while the DSM-IV and the *International Classification of Disease*-*10* (ICD-10) had the cutoff set at 7 years of age [3, 4]. Despite ADHD being labeled as a childhood disorder, a substantial proportion of those affected remain symptomatic well into adulthood [2]. ADHD is a major public health concern: ADHD and conduct disorder (CD) were the third worldwide leading psychiatric illness in disability-adjusted life years (DALY) in adolescents aged 10–19 years, behind depressive and anxiety disorders [5]; the median worldwide direct medical costs for children with ADHD were estimated to be 4306$ over 9 years compared to 1944$ for children without ADHD [6].

The worldwide pooled prevalence of ADHD has been estimated at 7.2%, keeping in mind that a minority of studies used randomized sampling and that there were significant regional differences [7]. Among the Arab countries of the Middle East, most of the prevalence studies have been undertaken in the Gulf region, as compared to the Levant region (Lebanon, Syria, Jordan, Palestine and Iraq), where only a handful were done: prevalence numbers ranged from 2.6% in Iraqi school children [8] to 11.8% in adolescents in Gaza [9]. These studies had several limitations as they relied on either self-questionnaires [8] or non-validated scales [9, 10]. The only previous ADHD prevalence study from Lebanon reported a prevalence of 3.2% among school children (aged 6–10 years), as evaluated by teachers’ scales [11]. The scarcity in research data is one of the major contributors to a lack of mental health resources in Lebanon (and in the region) as epidemiological evidence is usually the impetus behind developing mental health awareness and funding treatment resources [12]. The Beirut Epidemiological Investigation of the Psychiatric Status of Youth (BEI-PSY) is the first general population-based survey study to investigate the prevalence, correlates, and treatment seeking behaviors related to psychiatric disorders among adolescents in Lebanon [13]. The specific aims of this paper include: (1) examining the sociodemographic characteristics of adolescents with ADHD compared to adolescents with other psychiatric illnesses and healthy subjects; and (2) investigating the correlates of having the diagnosis of ADHD compared to not having the diagnosis. The results are discussed vis-à-vis regional and international literature, and evidence-informed recommendations are provided for future research and policy-making.

## Methods

### Sampling and data collection

BEI-PSY is a cross-sectional survey that targeted Arabic speaking adolescents, aged 11–17 years and 11 months, living in Beirut between March 2012 and December 2012. Recruitment was carried out using a multistage cluster sampling technique whereby neighborhoods, streets, and then households were sampled within Beirut. Sampling reflected the diversity in socioeconomic backgrounds in the city and cluster sizes were proportional to the population density in each segment area. The number of households approached in each segment area was divided equally between neighborhoods then among streets because there were no estimates for the number of households in each street and neighborhood. Households were considered eligible if they had at least one Arabic-speaking adolescent. Within eligible households, one adolescent was randomly selected and was interviewed along with a parent/legal guardian (preference given to mothers). Interviews were conducted by well-trained data collectors. Quality control was assured through call-backs of 10% of the recruited households selected at random to verify the accuracy of the retrieved information. Further details on the study protocol can be found elsewhere [13].

### Instruments and measures

Each adolescent and his/her parent/legal guardian were separately interviewed using the *development and well*-*being assessment* (DAWBA) [14]. Clinical diagnoses were generated based on the DAWBA by a child and adolescent psychiatrist and a licensed masters-level psychologist. All adolescents were asked to complete the *peer*-*relations questionnaire* (PRQ) [15] as well as the *strengths and difficulties questionnaire* (SDQ) [16]; the parent/legal guardian was also asked to fill out the SDQ as well as a questionnaire inquiring about the adolescent’s demographics, family and school situation, and psychiatric family history. Basic demographic information was collected from the parent and included information about the family size and income level, the adolescent and his/her parents’ educational level and the adolescent’s general health.

#### Development and well-being assessment

The DAWBA is a tool consisting of multiple questionnaires (with open-ended and closed-ended questions) addressed to the adolescent and a parent to help generate psychiatric diagnoses in children and adolescents based on the DSM-IV and the ICD-10. The questionnaire for ADHD focuses on the parent’s report of symptoms but also asks the parent about the teacher’s report on hyperactivity, poor attention and impulsivity. The information collected is then reviewed by a mental health professional to verify or overrule the generated diagnoses. Smoking, alcohol and substance use are also explored in the DAWBA through assessing frequency and intensity of use, functional impact and desire to quit. Cigarette smoking and alcohol use were finally analyzed as dichotomous (yes to any use versus no) in the past 4 weeks, since any underage smoking or drinking is noteworthy in this age group. In the present study, an Arabic version of the DAWBA was used [17]. This version was validated in a Lebanese clinical sample of children and adolescents showing excellent inter-rater reliability and substantial agreement against clinician diagnosis for disruptive disorders [18].

#### Peer-relations questionnaire

The PRQ is a 12-item, 4-point Likert scale (from 1 = Never to 4 = Very Often) with 3 sub-scores: bullying (PRQ-Bully), being victimized (PRQ-Victim), and pro-social behavior (PRQ-Prosocial). A translated, back-translated final Arabic version of the scale was found to be accurate showing good internal consistency in this sample [“PRQ-Victim” (α = 0.74) and “PRQ-Bullies” (α = 0.70)].

#### Treatment seeking attitude

Current (within the last 6 months) and past attitudes towards seeking mental health services (psychiatric or psychological) as well as any history of psychiatric treatment in any member of the family were also assessed using a questionnaire with open and closed-ended questions.

### Ethical considerations

BEI-PSY was approved by the Institutional Review Board (IRB) at the American University of Beirut. Written informed consents from participating parents/legal guardians and assents from adolescents were obtained. All participating families were given a referral list with addresses of mental health centers in the city. Participants were given a stationary kit at the end of their participation.

### Data analysis

To answer the first aim, sociodemographic characteristics, PRQ scores, SDQ Total Impact scores and treatment-seeking attitude measures were compared across three subgroups: adolescents with ADHD (ADHD), adolescents with a psychiatric diagnosis other than ADHD (psychiatric controls) and adolescents with no psychiatric disorders (Healthy). Pearson’s Chi square test and Fisher’s exact test were used to assess the bivariate association between two categorical variables. The Kruskal–Wallis H test and the Mann–Whitney U test were used to compare non-normally distributed continuous variables across all three subgroups.

To address the second aim, and specifically assess the medical and psychiatric correlates of a positive diagnosis of ADHD versus not having ADHD (whether diagnosed with another disorder or not), we used a multivariate logistic regression model adjusting for age, gender, nationality, household income, parental educational level and relationship status, as well as variables found to be statistically significant in bivariate analyses. The threshold for statistical significance was set at α = 0.05 based on two-tailed tests. When pairwise comparisons were done, the Bonferroni method was used to adjust for multiple comparisons and α was set accordingly. Analysis was conducted using the *statistical package for the social sciences* (SPSS) [version 22.0].

## Results

### Sociodemographic characteristics and clinical profiles

Among the total sample of 510, 52 (10.20%) were diagnosed with ADHD, of which around 77% had the combined type and 6% had the inattentive type. Table 1 compares the three subgroups [ADHD subgroup, adolescents diagnosed with a mental health disorder other than ADHD (i.e. psychiatric controls) and the healthy subgroup] on various sociodemographic characteristics. Among those diagnosed, 35 (67.31%) were males and 49 (94.23%) were of Lebanese nationality. There was a significant group difference in the parental relationship status (p = 0.004), in having the biological father (p = 0.043) and the biological mother (p = 0.021) residing at home, in having a positive psychiatric family history (p = 0.006), in school attendance (p = 0.022), in repeating at least one school grade (p = 0.003), in receiving special educational services (p = 0.021), and in receiving tutoring at home (p < 0.001). Post-hoc pairwise comparisons showed that, compared to the healthy subgroup, the ADHD subgroup had a significantly higher proportion of adolescents who had a positive psychiatric family history (15.38 vs 4%; p = 0.003), repeated at least one school grade (44.68 vs 27.74%; p = 0.003) and who were tutored at home (29.79 vs 11.57%; p = 0.002), while the psychiatric controls subgroup only differed on the proportion of those who had tutoring at home (27.40 vs 11.57%; p = 0.001). There were no significant pairwise comparisons between the ADHD and the psychiatric controls subgroups.

**Table 1:** Sociodemographic characteristics of ADHD subgroup as compared to psychiatric controls subgroup and healthy subgroup

Characteristic	ADHD (N = 52)		Psychiatric controls (N = 81)		Healthy (N = 377)		Total sample (N = 510)		Test statistic	p value
	Mean	SD	Mean	SD	Mean	SD	Mean	SD		
Age	13.67	2.06	13.84	2.11	13.99	2.16	13.94	2.14	H = 1.120	0.571
Mean household size	4.94	1.16	4.75	1.34	5.12	1.45	5.05	1.41	H = 4.481	0.093
	**N**	**%**	**N**	**%**	**N**	**%**	**N**	**%**		
Male gender	35	67.31	42	51.85	207	54.91	284	55.69	$\chi_{df = 2}^{2}$ = 3.421	0.181
Lebanese nationals	49	94.23	70	86.42	340	90.19	459	90.00	$\chi_{df = 2}^{2}$ = 2.202	0.332
Parents relationship status									FET = 16.919	0.004^†^
Married	46	88.46	66	81.48	355	94.16	467	91.57		
Separated	2	3.85	6	7.41	7	1.86	15	2.94		
Divorced	3	5.77	3	3.70	6	1.59	12	2.35		
One parent deceased	1	1.92	6	7.41	9	2.39	16	3.14		
Biological father residing at home	46	88.46	71	87.65	356	94.43	473	92.75	FET = 6.247	0.043ˆ
Biological mother residing at home	48	92.31	75	92.59	368	97.61	491	96.27	FET = 7.288	0.021ˆ
Highest parental educational level									FET = 7.917	0.406
Less than elementary	3	5.77	2	2.47	4	1.06	9	1.77		
Up to middle school	16	30.77	28	34.57	117	31.03	161	31.57		
High school degree	11	21.15	24	29.63	111	29.44	146	28.63		
University bachelor’s degree	17	32.69	22	27.16	117	31.03	156	30.59		
Master’s degree or above	5	9.62	5	6.17	28	7.43	38	7.45		
Total household monthly income (USD)									$\chi_{df = 4}^{2}$ = 4.541	0.338
233–800	20	39.22	36	44.44	126	33.78	182	36.04		
801–1600	21	41.18	34	41.98	167	44.77	222	43.96		
>1600	10	19.61	11	13.58	80	21.45	101	20.00		
Positive psychiatric family history	8*	15.38	5	6.25	15	4.00	28	5.49	FET = 9.387	0.006
School Attendance	47	90.38	75	90.12	363	96.29	483	94.71	FET = 7.248	0.022ˆ
Schooling									FET = 2.862	0.569
Formal public school	10	21.28	23	31.51	117	32.23	150	31.06		
Formal private school	36	76.60	47	64.38	231	63.64	314	65.01		
Repeated at least one school grade	21*	44.68	26	35.62	87	23.97	134	27.74	$\chi_{df = 2}^{2}$ = 11.566	0.003
Receives special education services	4	8.51	6	8.22	9	2.48	19	3.93	FET = 8.185	0.013ˆ
Receives tutoring at home	14*	29.79	20	27.40	42	11.57	76	15.73	$\chi_{df = 2}^{2}$ = 19.236	<0.001

*H* Kruskal–Wallis test, *FET* Fisher’s exact test, $\chi_{df = 2}^{2}$: Pearson chi square (*df* degrees of freedom)

^†^The proportions of married, separated, divorced and widowed parents were significantly different between the psychiatric controls subgroup and the healthy subgroup

ˆ Pairwise comparisons were non-significant after adjusting for multiple comparisons

* Significantly different than the healthy subgroup after adjusting for multiple comparisons

### Treatment seeking attitude

**Fig. 1:**
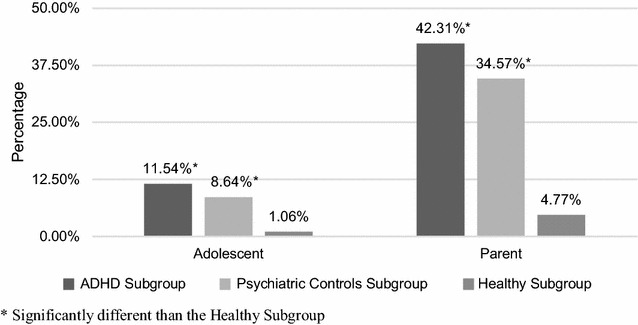
Treatment seeking attitudes across subgroups. Treatment seeking attitudes were compared across the three subgroups through the percentage of adolescents who were ever interested in seeking a mental health professional and the percentage of parents who ever considered taking their adolescent to see a mental health professional. The percentages of adolescents with ADHD and their parents who ever considered seeking professional help were significantly higher compared to their healthy counterparts

Adolescents and parents in the ADHD subgroup were more likely than adolescents and parents in the healthy subgroup to consider seeing a mental health professional [(11.54 vs 1.06%; p < 0.001) for adolescents and (42.31 vs 4.77%; p < 0.001) for parents respectively] but not more so than adolescents and parents in the psychiatric controls subgroup (Fig. 1). However, only three adolescents (5.77%) in the ADHD subgroup (eight in the entire sample) reported to have ever received any treatment for their condition.

### Peer relations

**Fig. 2:**
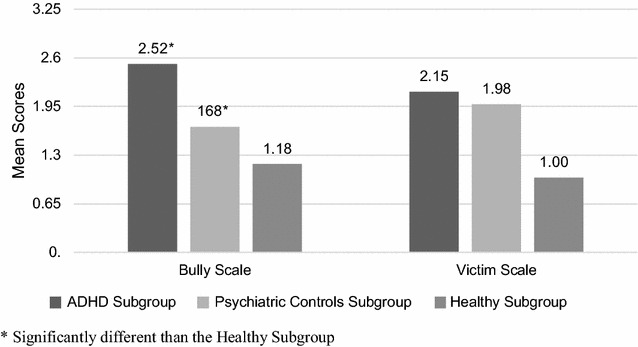
Peer relations questionnaire scores across subgroups. The peer relations questionnaire (PRQ) scores on the bully and victim subscales were compared across the three subgroups. Adolescents diagnosed with ADHD were significantly more likely to engage in bullying and be victims of bullying than healthy adolescents

There were significant differences among the three subgroups in the PRQ Bully subscale scores (Kruskal–Wallis’ Chi Square H: 13.503; p = 0.001) and Victim subscale scores (H: 26.629; p < 0.001). In pairwise comparisons, differences were significant between the ADHD and the healthy subgroups on the Bully subscale (Mann–Whitney U: 7288.5; p = 0.001) and the Victim subscale (U: 7181.0; p = 0.001) while the ADHD and the psychiatric controls subgroups did not differ on these two subscales (Fig. 2).

### Impact on the adolescent

**Fig. 3:**
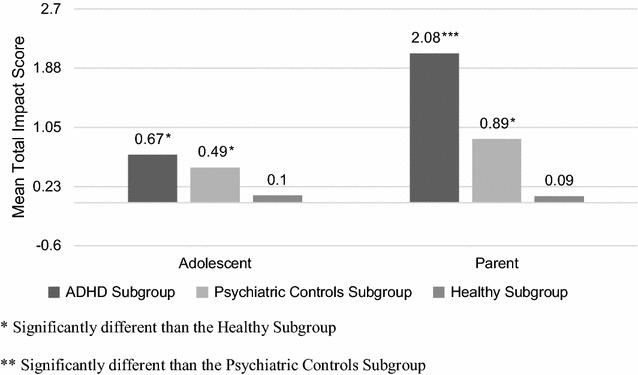
Mean total impact scores as measured on strengths and difficulties questionnaire across subgroups. The mean total impact scores as measured on strengths and difficulties questionnaire as per the adolescent and the parent’s report were compared across the three subgroups. Parents of adolescents with ADHD scored the total impact significantly higher than parents of adolescents in the remaining subgroups. Adolescents with ADHD scored the impact significantly higher than healthy adolescents

Impact scores as measured by the SDQ’s impact supplement significantly differed between the three subgroups, whether they were reported by the parents (H: 125.634; p < 0.001) or by the adolescents (H: 29.852; p < 0.001): scores were highest in the ADHD subgroup, followed by the psychiatric controls, then lowest among the healthy subgroup. Specifically, adolescents in the ADHD subgroup scored on average 2.08 on the parent’s report, compared to 0.89 in the psychiatric controls subgroup (U: 1408.0; p = 0.001, per pairwise comparison) and 0.09 in the healthy subgroup (U: 4321.5; p < 0.001, per pairwise comparison). On the adolescent’s report, the average total impact score in the ADHD subgroup was significantly higher compared to the healthy subgroup’s score (0.67 vs 0.10; U: 8495.5; p < 0.001) while it did not significantly differ with the psychiatric controls’ score (Fig. 3).

### Psychiatric comorbidities and correlates

Within the ADHD subgroup, 19 adolescents (36.54%) had a comorbid emotional disorder, 12 (23.08%) had oppositional defiant disorder (ODD) and 7 (13.46%) had CD, while 14 (26.92%) were reported to have a chronic medical condition. Cigarette smoking was more prevalent in the ADHD subgroup than in the psychiatric controls subgroup (19.23 vs 8.30%) as well as alcohol use (23.08 vs 10.26%).

After adjusting for age, gender, nationality, mean household income, parental educational level and relationship status, family psychiatric history, school attendance, repeating grades and receiving home tutoring in the multivariate logistic regression analysis, adolescents diagnosed with ADHD were found to be significantly more likely to have any emotional disorder [adjusted odds ratio (OR): 3.36; 95% CI 1.43–7.89] and ODD (adjusted OR: 71.79; 95% CI 12.67–406.66) and to drink alcohol (adjusted OR: 3.76; 95% CI 1.16–12.22) than adolescents not diagnosed with ADHD (Table 2).

**Table 2:** Prevalence of comorbidities in adolescents with ADHD versus those without

Comorbidity	ADHD subgroup (N = 52)		Non-ADHD subgroup (N = 458)		Adjusted odds ratio^a^	
	N	%	N	%	OR	95% CI
Any emotional disorder	19*	36.54	68	14.85	3.36	1.49–7.89
Oppositional defiant disorder	12*	23.08	3	0.01	71.79	12.67–406.66
Conduct disorder	7*	13.46	3	0.01	14.08	0.99–200.59
Any chronic medical condition	14*	26.92	66	14.41	2.07	0.87–4.95
Cigarette smoking	10*	19.23	38	8.30	1.08	0.24–4.86
Alcohol drinking	12*	23.08	47	10.26	3.76	1.16–12.22

Were included in the multivariate model 475 subjects who had no missing data

* Significantly different than the Non-ADHD subgroup based on bivariate Chi Square analysis (p < 0.05)

^a^Odds ratios were adjusted for age, gender, nationality, mean household income, parental educational level and relationship status, family psychiatric history, school attendance, repeating grades and receiving home tutoring

## Discussion

BEI-PSY is the first study to investigate the prevalence of psychiatric disorders among adolescents residing in Beirut, and its findings highlight a high prevalence of ADHD among adolescents residing in that area. Compared to healthy controls, adolescents with ADHD were more likely to be associated with a family history of psychiatric illness, a personal history of chronic medical illness, alcohol use, bullying and being bullied, as well as a lower school performance and an increased reliance on home tutoring. A diagnosis of ADHD was highly comorbid with emotional disorders, and ODD or CD. Parents of adolescents with ADHD reported a significantly higher impact of the illness compared to parents of adolescents in the other two groups. However, attitudes towards treatment seeking were similar regardless of the psychiatric diagnosis and only a minority of parents ever sought mental health services.

The prevalence of ADHD in this study is 10.2% and is relatively high compared to prevalence estimates reported in other parts of the Arab world [19] and in an earlier Lebanese study [11] which found a prevalence of 3.2% among school-aged children using a teacher rating scale. Our high prevalence could be explained by a difference in methodology as our study used a structured diagnostic tool to interview both adolescents and their parents in order to make a diagnosis based on DSM-IV criteria, whereas most other regional studies were conducted in school samples, surveyed pre-adolescent children, and relied on self-questionnaires or teachers’ rating scales [19, 20]. Furthermore, although our sample was socioeconomically diverse, our sampling area was uniformly urban, and studies conducted in urban environments have been associated with increased parental reporting of ADHD [21]. The DAWBA has been shown to be highly accurate and reliable in detecting childhood psychiatric disorders, including ADHD [14, 22] and our prevalence and male-to-female ratio are similar to the numbers found in the 2011 USA National Survey of Children’s Health for children aged between 11 and 17 years [23].

Adolescents diagnosed with ADHD had distinct characteristics when compared to healthy adolescents and adolescents with other psychiatric diagnoses. Previous studies in the Arab region found that being raised by a single parent [24], having polygamous parents [25], having a parent with history of ADHD [26] and having a low socioeconomic level [27] were associated with being diagnosed with ADHD. In our study, we found that ADHD was associated with a positive family psychiatric history as reported by the parents who were interviewed; however, we did not find an association between having ADHD and the parents’ marriage status and socioeconomic level. It has been consistently reported in the literature that parents of children with ADHD are more likely to have psychiatric illnesses such as ADHD, mood and anxiety disorders, personality disorders and substance use [28, 29]. Contrary to our psychiatric controls subgroup, our ADHD subgroup had a significantly poorer academic performance (higher propensity to repeat grades and to need home tutoring) compared to the healthy subgroup, a finding that has been well-documented in the international literature. A recent meta-analysis found that poor attention and hyperactivity are strong predictors of academic problems such as repeating grades and using special education services even after adjusting for IQ, socioeconomic status and comorbidities [30]. Added to the academic difficulties, the ADHD and the psychiatric controls subgroups experienced more peer-relation difficulties than the healthy subgroup, as evidenced by a higher propensity to bully others and to be bullied. Indeed, it has been shown that school children who have any type of mental health issues (including ADHD) were more likely to be involved in bullying as perpetrators and/or victims than healthy school children [31, 32].

Similar to international literature, adolescents in our ADHD subgroup had significantly more psychiatric comorbidities such as emotional disorders and ODD [33, 34], and more alcohol use [35] as compared to those who do not have ADHD. The loss of other significant associations (e.g., with CD, chronic medical conditions, and cigarette smoking) might be due to the small numbers as evidenced by the large confidence intervals of the odds ratios.

Although all psychiatric illnesses require in their diagnostic criteria impairment in functioning, our study showed a significantly higher burden of disease for ADHD on the adolescent and his/her surroundings as measured by the total impact score on the SDQ, compared to other psychiatric illnesses. However, there seems to be a discrepancy between the parents’ and the adolescents’ perceptions of burden of ADHD: parents found it to be significantly burdensome as evidenced by an average total impact score above 2, whereas affected adolescents had an average score of 0.67, reflecting a perception of a lack of impairment [36]. Our results are in line with previous reports highlighting the clinical importance of parent-reported impact of illness: it was found to be predictive of new-onset seeking of mental health services and new-onset of self-harm, whereas self-reported impact was not [37]. In our study, the higher burden of disease in ADHD was indeed associated with a better awareness to consider seeking help from a mental health professional; however, only a negligible proportion of those actually sought it. A similar reluctance to seek psychiatric treatment has been reported in Lebanese adults (only 10.9% of diagnosed adults obtained treatment) and has been explained by the preponderance of barriers to treatment in Lebanese society, including financial constraints and a lack of mental health awareness [38]. The international literature has consistently reported that ADHD is undertreated [39, 40] and a recent meta-analysis identified the following main barriers to seeking treatment for ADHD: child characteristics (sex, age, ethnicity, comorbidities), family’s socioeconomic status, structural barriers (financial costs, healthcare system), parents’ perception of ADHD and of its treatment and fear of stigma [41]. Further research exploring Lebanese-specific barriers to seeking mental health services in general and ADHD treatment in particular is timely.

### Limitations and offsetting strengths

The findings of this study must be interpreted with some limitations in mind. Our findings may not be generalizable to the entire population of Lebanon since our sampling was strictly limited to Beirut, given the lack of an updated official population census (last one conducted in 1932). Our sample, however, reliably reflected the diversity of the socioeconomic strata of the Lebanese society as a whole [42]. In addition, we administered the SDQ to the adolescent and one parent/legal guardian but not to a teacher, which might have underestimated the impact of the illnesses surveyed.

Despite these limitations, this study is the first to investigate ADHD in adolescents in Lebanon and the first in the Arab region to uncover the significant medical, academic and functional burden as well as important correlates. Our findings highlight the need for relevant governmental authorities and mental health advocates to further develop public awareness about the symptoms of ADHD and the functional impact of the illness and the availability of resources for treatment. These efforts should specifically focus on schools as teachers and counselors can be educated to detect possible symptoms and discuss with the parents referrals for an assessment. Finally, mental health specialists should be sensitized to extensively discuss treatment options and their benefits and risks not just with the parents, but also with the affected adolescent.

## Abbreviations

ADHD: attention-deficit/hyperactivity disorder
BEI-PSY: Beirut Epidemiological Investigation of the Psychiatric Status of Youth
CD: conduct disorder
COI: cost-of-illness
DALY: disability-adjusted life years
DAWBA: development and well-being assessment
DSM: Diagnostic and Statistical Manual
ICD: International Classification of Disease
IQ: intelligence quotient
IRB: Institutional Review Board
ODD: oppositional defiant disorder
OR: odds ratio
PRQ: peer-relations questionnaire
SDQ: strengths and difficulties questionnaire
SPSS: statistical package for the social sciences
USA: United States of America
